# Potential of bacteriophage ΦAB2 as an environmental biocontrol agent for the control of multidrug-resistant *Acinetobacter baumannii*

**DOI:** 10.1186/1471-2180-13-154

**Published:** 2013-07-08

**Authors:** Li-Kuang Chen, Yu-Lin Liu, Anren Hu, Kai-Chih Chang, Nien-Tsung Lin, Meng-Jiun Lai, Chun-Chieh Tseng

**Affiliations:** 1 Institute of Medical Sciences, Department of Laboratory Diagnostics, College of Medicine, Tzu Chi University, Hualien, Taiwan; 2 Department of Laboratory Medicine, Clinical Pathology, Emerging Infectious Pathogen Research Laboratory, Buddhist Tzu Chi General Hospital, Hualien, Taiwan; 3 Department and Graduate Institute of Public Health, Tzu Chi University, Hualien, Taiwan; 4 Department of Laboratory Medicine and Biotechnology, Tzu Chi University, Hualien, Taiwan; 5 Master Program, Microbiology, Immunology, and Biochemistry, School of Medicine, Tzu Chi University, Hualien, Taiwan

**Keywords:** Bacteriophage, Biocontrol, MDRAB

## Abstract

**Background:**

Multidrug-resistant *Acinetobacter baumannii* (MDRAB) is associated with nosocomial infections worldwide. To date, the use of a phage to prevent infections caused by MDRAB has not been demonstrated.

**Results:**

The MDRAB-specific phage ϕAB2 was stable at 4°C and pH 7 in 0.5% chloroform solution, and showed a slight decrease in plaque-forming units (PFU)/ml of 0.3–0.9 log after 330 days of storage. The addition of ϕAB2 at a concentration of at least 10^5^ PFU/ml to an *A. baumannii* M3237 suspension killed >99.9% of *A. baumannii* M3237 after 5 min, regardless of *A. baumannii* M3237 concentration (10^4^, 10^5^, or 10^6^ colony-forming units (CFU)/ml). The addition of ϕAB2 at a concentration of 10^8^ PFU/slide (>10^7^ PFU/cm^2^) to glass slides containing *A. baumannii* M3237 at 10^4^, 10^5^, or 10^6^ CFU/slide, significantly reduced bacterial numbers by 93%, 97%, and 99%, respectively. Thus, this concentration is recommended for decontamination of glass surfaces. Moreover, infusion of ϕAB2 into 10% glycerol exhibited strong anti-MDRAB activity (99.9% reduction), even after 90 days of storage. Treatment of a 10% paraffin oil-based lotion with ϕAB2 significantly reduced (99%) *A. baumannii* M3237 after 1 day of storage. However, ϕAB2 had no activity in the lotion after 1 month of storage.

**Conclusions:**

Phages may be useful for reducing MDRAB contamination in liquid suspensions or on hard surfaces. Phages may also be inoculated into a solution to produce an antiseptic hand wash. However, the phage concentration and incubation time (the duration of phage contact with bacteria) should be carefully considered to reduce the risk of MDRAB contamination.

## Background

*Acinetobacter baumannii* is a Gram-negative coccobacillus frequently associated with nosocomial infections worldwide [1,2]. It is an opportunistic pathogen with a wide spectrum of clinical manifestations, including pneumonia, meningitis, and blood stream, urinary tract, and wound infections [3,4]. *A. baumannii* has developed resistance to broad-spectrum antibiotics and has thus become problematic in intensive care units (ICUs) [5]. In Taiwan, the first multidrug-resistant *A. baumannii* (MDRAB) strain was identified in 1998 [6]. This strain was among the top three pathogens causing nosocomial infections in ICUs in Taiwan from 2003–2009 [7], and the incidence of MDRAB infections worldwide has continued to increase [5,8-10].

Nosocomial MDRAB infections are usually transmitted between patients by contaminated health-care personnel [11]. Therefore, there is growing interest in controlling the spread of MDRAB caused by health-care workers, contaminated equipment, and ICU environments through disinfection methods. To date, several disinfection techniques have been evaluated for inactivating *A. baumannii*, including pasteurization [12], ultraviolet light [13], chemical sanitizers [14-16], ozone [17], and photocatalysis [18]. These sterilization techniques are highly effective in reducing *A. baumannii* contamination, but may be harmful to humans or surface materials in the ICU environment. Moreover, extensive use of chemicals can cause bacteria to develop resistance to chemical sanitizers [16,19]. For example, the growth and virulence of MDRAB are enhanced following exposure to ethanol and alcohol-based hand rubs [20]. Thus, there is an immediate need to develop alternative strategies for preventing the spread of MDRAB.

Bacteriophages (phages) are natural parasites of bacteria and are extremely host-specific. Therefore, the use of phages to reduce the concentration of specific bacterial foodborne pathogens has gained increasing attention [21-24]. For example, phages have been used to treat foods contaminated with strains of *Campylobacter*[22], *Enterobacter*[25], *Escherichia coli* O157 [26], *Listeria*[23], *Salmonella*[27], and *Staphylococcus*[28,29]*.* The levels of these bacterial pathogens have been successfully reduced by 1–5 logs, depending upon the method used. Moreover, the United States Food and Drug Administration has already approved the use of a *Listeria*-specific phage, Listex P100, for food preservation [30]. Although these studies suggest that bacteriophages might be highly effective in reducing MDRAB levels, this has not been studied in detail.

Although phages can significantly reduce the amount of pathogenic bacteria in liquid foods [22-24], the use of phages to reduce the levels of bacteria on hard surfaces has rarely been studied. Culture-positive swab samples of MDRAB have been recovered from frequently touched surfaces in ICUs [14,31]. These observations indicate the possible role of environmental surfaces in the spread of MDRAB [32]. Liquid suspensions containing a high concentration of phages allow the free diffusion of phages to ensure contact with their specific host [23]. However, for hard surfaces, an uneven and large surface area may limit the distribution of phage particles and decrease their ability to reach their bacterial targets [26]. This is especially true for low concentrations of bacteria that are unevenly distributed in the environment [33]. Therefore, the effects of phage concentration, host cell concentration and incubation time (the duration of phage contact with bacteria) on the degree of biocontrol on hard surfaces should be further investigated.

Despite intensive programs to encourage hand washing by health-care personnel, there is still a high rate of MDRAB transmission in ICUs. O’Flaherty [34] demonstrated the inclusion of phage K in an oil-based cream killed *Staphylococcus aureus* on agar and in broth cultures. Thus, a phage-containing hand cream could reduce pathogenic bacteria [34]. However, that study did not report on the stability of phages in the cream or on the exact degree of the bactericidal effect achieved. If a phage-containing cream were feasible for infection control, this approach would likely reduce the transmission of MDRAB from the hands of health-care personnel to patients in ICUs.

The first lytic phage shown to specifically infect MDRAB was characterized in 2010 [35] and belonged to the *Podoviridae* family, with a broad host range amongst MDRAB strains. This is the only known phage capable of infecting *A. baumannii* ATCC17978, whose genome has been fully sequenced [35]. In addition, ϕAB2 can rapidly adsorb to its host and has a large burst size [35]. These advantages make ϕAB2 a good model phage for controlling the prevalence of nosocomial infections caused by MDRAB. To our knowledge, most biocontrol studies have focused on using phages as food decontaminants [21,23,26,36,37]. The application of a phage as a disinfectant agent for the control of MDRAB has not been previously reported. Consequently, this study aimed to evaluate the ability of ϕAB2 phage to reduce MDRAB in suspension and on experimentally-contaminated glass surfaces. In addition, the ability of ϕAB2 in a paraffin oil-based lotion or glycerol to reduce the number of viable MDRAB was determined. The stability of ϕAB2 under different environments (temperature, pH, chloroform, and glass surface) was also evaluated.

## Results

### Adsorption and one-step growth curve of ϕAB2

**Figure 1:**
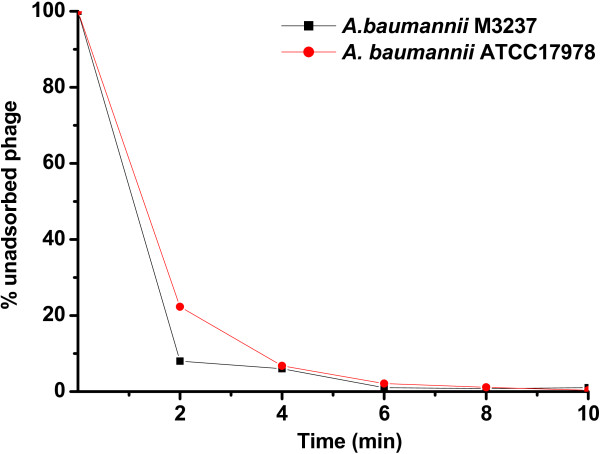
**Adsorption of ϕAB2 to *A. baumannii *M3237 and *A. baumannii *ATCC 17978.** Approximately 95% of the phage particles were adsorbed onto the cells at 5 min and 100% were adsorbed at 10 min post-infection.

ϕAB2 rapidly was adsorbed onto both *A. baumannii* M3237 and *A. baumannii* ATCC 17978 (Figure 1). Within 5 min, greater than 95% of the phage particles were adsorbed to *A. baumannii* M3237 and *A. baumannii* ATCC 17978, and nearly 100% were adsorbed by 10 min.

### Effect of temperature on ϕAB2 stability

**Figure 2:**
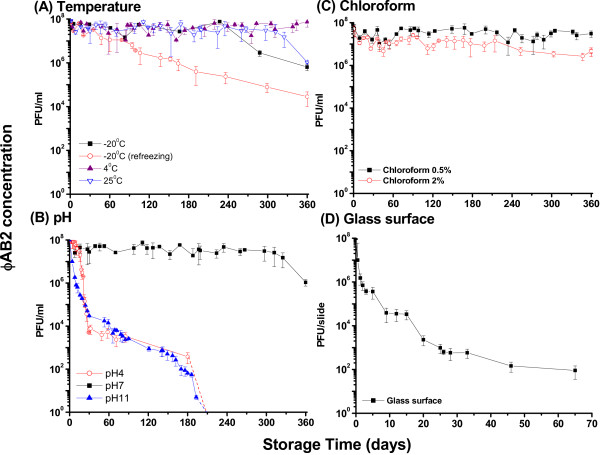
**Stability of ϕAB2 under (A) temperature, (B) pH, (C) chloroform, and (D) glass surface.** The dotted line indicates no plaque survival at the respective storage time. These experiments were repeated three times and the data shown are the mean ± SEM.

Figure 2A shows the stability of ϕAB2 stored in deionized water at −20°C, 4°C, and 25°C, over 360 days. When the phages were stored in deionized water at −20°C, 25°C, and 4°C for 360 days they retained 0.6%, 1.0%, and 66.0% of infectivity, respectively. Although ϕAB2 had infectivity retention of more than 50% when stored in deionized water after 360 days at 4°C, infectivity retention of more than 50% was only observed up to 220 days in samples stored at −20°C or 25°C. The effect of refreezing on phage survival demonstrated that ϕAB2 was unstable when the sample was frozen repeatedly, as greater than 99.9% of phages lost infectivity after refreezing during a 360-day storage period at −20°C.

### Effect of pH on ϕAB2 stability

The optimal pH for ϕAB2 stability was determined (Figure 2B). ϕAB2 was relatively stable following 360-day incubation at pH 7. Under these conditions, there was a 2-log decrease in ϕAB2 phage titers from the initial titer of 10^8^ PFU/ml. However, ϕAB2 titers decreased by over 5-logs after 180-day incubation at pH 4 or pH 11. In extremely acidic conditions, at pH 2, no ϕAB2 plaques were identified after 10 min (data not shown). Thus, ϕAB2 is unstable under extreme pH conditions.

### Effect of chloroform concentration on ϕAB2 stability

ϕAB2 titers were reduced following exposure to chloroform concentrations of 0.5% and 2% (Figure 2C). For phage purification, a chloroform concentration of 0.5–2% (v/v) is typically used, thus the infectivity of ϕAB2 following exposure to 0.5% and 2% chloroform was investigated. ϕAB2 exposed to 0.5% chloroform retained stable infectivity of greater than 20% following a 360-day storage period. However, infectivity retention of ϕAB2 was only 5% following a 360-day storage period in 2% chloroform (Figure 2C).

### ϕAB2 stability on glass slides

Desiccation reduced the stability of ϕAB2 when spiked onto a glass surface over a 65-day period (Figure 2D). There was a 1-log decrease in ϕAB2 titers (initial phage concentration of 10^8^ PFU/slide) after 12 h on the glass surface. Infectivity of ϕAB2 on a glass slide was 0.1% after 7 days and 0.001% after 30 days. Thus, ϕAB2 could survive on a dried glass surface for 2 months, although a large reduction in ϕAB2 titers was observed.

### Reduction of MDRAB by ϕAB2 in a liquid suspension

**Figure 3:**
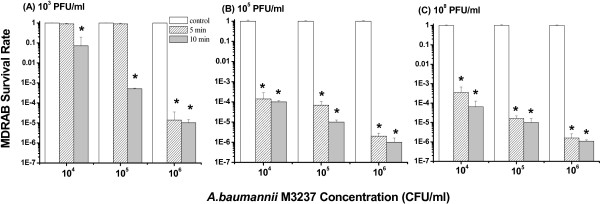
**Bactericidal effect of different concentrations: (A) 10**^**3 **^**(B) 10**^**5**^, **and (C) 10**^**8 **^**PFU/ml of ϕAB2 on different concentrations of *A. baumannii *M3237 in a liquid suspension, at incubation times of 5 and 10 min.** The survival rate was calculated as in the Methods section. These experiments were repeated three times, and the data shown are the mean ± SEM. *p < 0.05 compared with the respective control group.

We next assessed the ability of ϕAB2 to reduce the concentration of *A. baumannii* M3237 in sterile water over different incubation times (the duration of contact of the phages with the hosts). The addition of ϕAB2 to a liquid suspension of *A. baumannii* M3237 had a strong bactericidal effect in all test groups except the 5 min incubation low dose group (10^3^ PFU/ml) (Figure 3). The ϕAB2 bactericidal effect showed a dose-response as the lowest concentration of ϕAB2 tested (10^3^ PFU/ml) exhibited the weakest bactericidal capability, which was 6,600-fold lower than when higher phage concentrations (10^5^ and 10^8^ PFU/ml) were used (Figure 3A). The addition of 10^5^ or 10^8^ PFU/ml ϕAB2 reduced the number of *A. baumannii* M3237 by at least 3-logs at all bacterial test concentrations after 5 min. After 10 min incubation, the effect was even greater, with at least a 4-log reduction in MDRAB survival rates (Figure 3B and C). In addition, the mean reduction in bacteria was greater when a higher initial bacterial concentration was used. Thus, the same concentration of ϕAB2 had a stronger bactericidal effect when added to high bacterial concentrations (10^5^ and 10^6^ CFU/ml) compared with low bacterial concentrations (10^4^ CFU/ml). All control groups showed a 100% survival rate. In addition to the phage and bacterial host concentrations, the incubation time was also important for the bactericidal effect. Approximately 95% of phage particles adsorbed to host cells within 5 min, and nearly 100% were adsorbed by 10 min (Figure 1). Therefore, we selected the 5 and 10 min time points to test the bactericidal effect of ϕAB2 in suspension. At a low phage concentration (10^3^ PFU/ml), an increase in the incubation time from 5 to 10 min resulted in a mean decrease of survival rate of MDRAB between 1.5- and 1,700-fold. In contrast, at higher phage concentrations (10^5^ PFU/ml and 10^8^ PFU/ml) there was a mean reduction of bacterial concentration of 1.4- to 7-fold when the incubation time was increased from 5 to 10 min.

### Bactericidal effect of ϕAB2 on a hard surface

**Figure 4:**
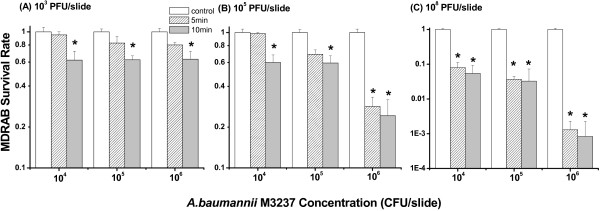
**Bactericidal effect of different concentrations: (A) 10**^**3 **^**(B) 10**^**5**^, **and (C) 10**^**8 **^**PFU/slide of ϕAB2 on different concentrations of *A. baumannii *M3237 on a glass surface following incubation times of 5 and 10 min.** The survival rate was calculated as in the Methods section. These experiments were repeated three times, and the data shown are the mean ± SEM. *p < 0.05 compared with respective control group.

The addition of ϕAB2 to a hard glass surface contaminated with *A. baumannii* M3237 had a bactericidal effect under some conditions (Figure 4). Phage concentrations of 10^3^ and 10^5^ PFU/slide caused a significant reduction (*p <* 0.05*,* 40% reduction) of *A. baumannii* M3237 cells (10^4^ and 10^5^ CFU/slide) after 10 min (Figure 4A and B). When a phage concentration of 10^8^ PFU/slide was used, the number of *A. baumannii* M3237 was significantly reduced (*p <* 0.05*,* >90% reduction) after 5 or 10 min for all concentrations of bacteria tested (Figure 4C). However, the bactericidal effect of ϕAB2 at 10^8^ PFU/slide was significantly lower for *A. baumannii* M3237 at 10^4^ and 10^5^ CFU/slide than at 10^6^ CFU/slide (*p* < 0.05). To date, there is no standard method for evaluating phage biocontrol efficiency on a hard surface. Incubation times of 5 and 10 min were chosen for surface tests on the basis of ϕAB2 adsorption data (Figure 1) and a previous study by Abuladze *et al*. [26]. Extending the incubation time from 5 to 10 min increased the mean bactericidal effect on *A. baumannii* M3237 1.3-fold under all test conditions.

### Use of ϕAB2 as a hand sanitizer in a paraffin oil-based lotion

**Figure 5:**
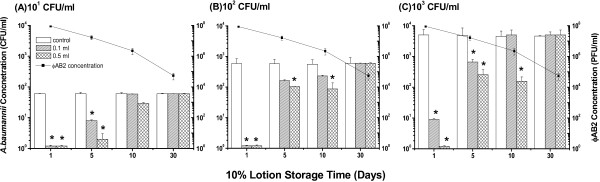
**Bactericidal effect of 0.1 ml and 0.5 ml of ϕAB2-containing lotion (stored up to 30 days) on different concentrations: (A) 10**^**1 **^**(B) 10**^**2**^, **and (C) 10**^**3 **^**CFU/ml of *A. baumannii *M3237 contaminated agar.** Phage titers (■) are shown on the right on the logarithmic scale. *p < 0.05 compared with the respective control group.

The stability of ϕAB2 in a lotion and its ability to kill *A. baumannii* M3237 when spread on agar (at a volume of 0.1 ml), were evaluated to determine the potential of ϕAB2 as a hand lotion antiseptic. Prior to the addition of the phage lotion, lysogeny broth (LB) agar was pre-contaminated with approximately 5 × 10^1^, 5 × 10^2^, or 5 × 10^3^ CFU/ml (coefficient variation % (CV%) = 3.0%) of *A. baumannii* M3237 (Figure 5). The initial phage concentration in the lotion was 10^8^ PFU/ml; however, this concentration decreased by approximately 98% after 10 days of storage (*p* < 0.05). Phage lotion stored for 1 day significantly reduced (*p* < 0.05) viable *A. baumannii* M3237 at initial concentrations of 10^1^, 10^2^ and 10^3^ CFU/ml on agar, by 97.6%, 99.8%, and 99.9%, respectively. Lotion stored for 5 days also significantly reduced (*p* < 0.05) the concentration of viable *A. baumannii* M3237 by 92%, 88%, and 90%, respectively. Lotion stored for longer than 5 days could not effectively reduce the *A. baumannii* M3237 concentration. Spreading a larger volume (0.5 ml) of lotion on agar did not significantly alter the number of *A. baumannii* M3237 killed by the phage, as compared with a smaller volume (0.1 ml).

### Use of ϕAB2 as a hand sanitizer in glycerol

**Figure 6:**
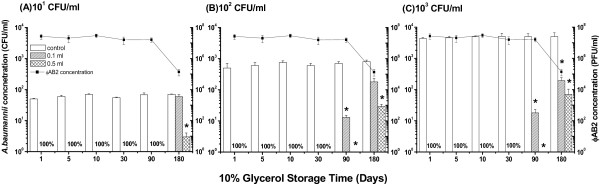
**Bactericidal effect of 0.1 ml and 0.5 ml of ϕAB2-containing glycerol (stored up to 180 days) on different concentrations: (A) 10**^**1 **^**(B) 10**^**2**^, **and (C) 10**^**3 **^**CFU/ml of *A. baumannii *M3237 contaminated agar.** Phage titers (■) are shown on the right on the logarithmic scale. *p < 0.05 compared with the respective control group. “100%” indicates 100% reduction in *A. baumannii* M3237 following application of either 0.1 or 0.5 ml of ϕAB2-containing glycerol.

Glycerol is used by the cosmetics industry to retain moisture in the skin. Therefore, the addition of ϕAB2 to glycerol may be an effective way to formulate a hand sanitizer that can decrease MDRAB contamination and retain moisture within the skin. Because the amount of glycerol in cosmetic products varies (usually less than 20%), a concentration of 10% (v/v) glycerol was evaluated in this study. Prior to the addition of the phage-containing glycerol, LB agar was pre-contaminated with approximately 5 × 10^1^, 5 × 10^2^, or 5 × 10^3^ CFU/ml (CV% = 12.3%) of *A. baumannii* M3237 (Figure 6). The ϕAB2 phage concentration (10^8^ PFU/ml) did not significantly decrease (less than a 1-log decrease) when added to a glycerol solution and stored for 90 days. The application of phage-containing glycerol stored for 90 days to inoculated agar significantly reduced (*p* < 0.05) the mean concentration of viable *A. baumannii* M3237 by 99.9%, regardless of the initial bacterial concentration. After 180 days of storage, ϕAB2 titers were decreased by approximately 2-logs (*p* < 0.05). The application of phage-containing glycerol stored for 180 days reduced the mean concentration of viable *A. baumannii* M3237 by 62.4%, 86.2%, and 98.6% when the initial concentration of *A. baumannii* M3237 was 10^1^ CFU/ml, 10^2^ CFU/ml, and 10^3^ CFU/ml, respectively. Similar to the effect observed with the lotion, the bactericidal effect of spreading a larger volume (0.5 ml) of the phage-containing glycerol on agar was not significantly different from that of a smaller volume (0.1 ml).

## Discussion

To date, most biocontrol studies have used phages for the decontamination of food and limited data are available concerning the stability of phages in an environmental matrix. Furthermore, the use of a phage to prevent infections caused by MDRAB has not been demonstrated. The ϕAB2 phage was selected as a model phage for this study because its DNA and protein profiles were previously determined [35]. The current study demonstrated that phages such as the ϕAB2 phage might be useful for reducing MDRAB contamination in liquid suspensions or on hard surfaces such as may be encountered in ICUs, and may be added to a solution to produce an antiseptic hand wash.

One issue with the human use of phages is their potential toxicity. Previously, we demonstrated ϕAB2 had 91–99% DNA sequence identity with the fully sequenced ϕAB1 and that to date, no putative or confirmed toxin genes have been identified in ϕAB2 [38]. In addition, no prophage-related genes were observed in ϕAB1, although Vallenet *et al*. suggested that putative prophage sequences account for 5.1% and 6.7% of the genomes of both *A. baumannii* strains [39]. Thus, it is reasonable to assume that ϕAB2 has no toxin genes or prophage-related genes, and we predict there will no safety issues related to toxin production or chromosomal integration of ϕAB2.

There have been limited studies regarding environmental effects on phage stability. A previous study investigated another *A. baumannii*-specific phage, AB1, which is relatively heat resistant and can survive temperatures of 50–60°C, and even a 15-min incubation at 90°C [40]. The stability of ϕAB2 at extremely high temperatures was not evaluated in the present study because ϕAB2 is proposed for use as an alternative sanitizer, so information regarding its stability for long storage periods at refrigerated or freezing temperatures was more relevant. Our study demonstrated that phage infectivity is strongly dependent on environmental conditions such as temperature, pH, and the presence of other organic substances. Investigation of the optimal pH for maintaining ϕAB2 infectivity demonstrated that the least damaging pH tested was pH 7, similar to the sewage from which ϕAB2 was isolated (pH 7.8). Yang *et al*. also demonstrated that the AB1 phage was most stable at pH 6, and that less than 42.9% of AB1 phages lost their infectivity in a range between pH 5–9 [40]. Thus, our environmental stability results indicated that ϕAB2 should be stored in a pH 7 solution at 4°C for extended storage if ϕAB2 is incorporated into a detergent.

Because MDRAB survives for long periods on environmental surfaces and may promote cross-transmission, we investigated the efficiency of ϕAB2 in reducing *A. baumannii* M3237 contamination on surfaces. We observed the ϕAB2 concentration required to reduce *A. baumannii* M3237 contamination was lower for liquid suspensions than hard surfaces. The mean survival rate ratio of *A. baumannii* M3237 between surface and liquid suspension ranged from 2–10,151 depending on the phage concentration. As ϕAB2 does not diffuse as freely on a hard surface as in a suspension, a higher concentration of ϕAB2 was required for surface decontamination of MDRAB compared with in solution.

The ability of phages to persist on a surface for extended periods is limited by many factors, such as desiccation [37], which may explain the loss of ϕAB2 infectivity after 2 months storage on a glass surface. Because ϕAB2 cannot survive for long periods on a hard surface, the phage detergent must be frequently re-applied to surfaces to provide persistent bactericidal or MDRAB activity.

Previous biocontrol studies suggested that high phage numbers should be used without relying on phage amplification [22,23]. Although ϕAB2 has a larger burst size than other phages [23,35], it is important to determine the optimal phage concentration that will allow efficient phage attachment and amplification for the quantity of MDRAB present. Experiments on environmental ICU samples have identified *A. baumannii* on 39% of the sampled surfaces with a mean *A. baumannii* DNA concentration of 19,696 copies [39]. Based on the results of our surface evaluation, we recommend that a phage concentration of at least 10^7^ PFU/cm^2^ be applied to surfaces in ICUs. This approach may not be suitable for the treatment of large surfaces, but may be useful for small biomedical devices. Abuladze *et al*. suggested a glass matrix is easier to decontaminate than gypsum [26]. Thus, the phage decontamination efficiency for different surfaces such as gypsum, plastic, Teflon, or other polymers may vary, and requires further investigation. In addition to phage concentration, the incubation time is also critical for surface applications. When a high phage concentration (10^8^ PFU/slide) was used to treat a surface contaminated with bacteria at a concentration of 10^5^ CFU/slide, an incubation time of 5 min resulted in a 96% reduction of *A. baumannii* M3237 numbers. This incubation time was caused a 94% reduction in the number of *Escherichia coli* O157:H7 [26] under the same test conditions.

MDRAB can be transmitted via the hands of health-care personnel. However, frequent or improper hand washing can cause skin to lose moisture or become irritated, reducing the hand washing rate despite intensive hand washing educational programs. Therefore, the addition of paraffin oil and glycerol to the formulation of antiseptic hand wash might retain skin moisture and reduce the transmission of MDRAB from the hands of health-care workers to patients. Alcohol-based hand rubs could reduce skin irritation [41] and reduce the number of bacteria more effectively than soap and water in a number of experimental models [42,43]. However, *A. baumannii* may metabolize low levels of alcohol to become more virulent [20]. Thus, an alternative hand washing approach is required to prevent microorganisms becoming tolerant to alcohol-based disinfectants in the future.

In this study, we designed two antiseptic hand wash experiments and observed a difference in the bactericidal effect between phage-containing lotion and glycerol solution, possibly related to the stability of ϕAB2 in different media. Because the detailed compositions of commercial creams are proprietary, it is difficult to explain the unpredictable changes of phage numbers in the cream, as phages could aggregate, disaggregate, or decay after long storage periods. O’Flaherty *et al*. demonstrated that *S. aureus*-specific phage K exhibited antibacterial activity when incorporated into a bismuth-based cream [34]. The bismuth cream exhibited well antibacterial activity, but the related phage stability was not reported. In contrast, we observed that ϕAB2 was stable in 10% glycerol after 90 days storage at room temperature. Glycerol is a common cryoprotectant for phage infectivity during storage at temperatures between −20 and −70°C. Other phages, including F-specific RNA bacteriophages, and *Bacteroides fragilis*-specific phages, are also stable in 10% glycerol for up to 50 days [44] and can retain their infectivity with even longer storage times.

## Conclusions

Since the introduction of antibiotics for clinical use, antibiotic-resistant bacteria, such as MDRAB, have emerged as important nosocomial pathogens worldwide. Our study used ϕAB2 as a model phage to demonstrate its potential for the prevention of nosocomial MDRAB infections. As MDRAB are resistant to almost all currently available antibiotics and sanitizers, phages represent an alternative environmental decontamination approach. Although some studies have focused on isolating and characterizing new phages with a broader host range, further information regarding the stability of phages in different environments is required before these phages are used in hospitals. While phages could be used to decontaminate environmental surfaces naturally contaminated by MDRAB, when bacterial cell numbers are low and the surface area is large, a high phage concentration (>10^7^ PFU/cm^2^) is required to ensure contact between phages and their hosts. This study demonstrated that high concentrations of phages might be inoculated into a lotion or glycerol and used as an antiseptic hand wash. However, the phage concentration and incubation time should be carefully determined to identify the optimal bactericidal effect on MDRAB.

## Methods

### Bacterial host strain and culture

We used *A. baumannii* M3237 as a bacterial host, instead of an antibiotic-sensitive strain such as *A. baumannii* ATCC 17978, for practical simulation of the bactericidal effect of ϕAB2 on MDRAB in a hospital environment. *A. baumannii* M3237was purchased from the Bioresource Collection and Research Center of Taiwan (BCRC 80276). *A. baumannii* M3237 is a MDRAB clinical isolate from the Buddhist Tzu-Chi General Hospital and was maintained and grown in LB or agar at 37°C.

### Phage preparation

ϕAB2 was isolated from the raw sewage of a local hospital [35]. A high-titer stock of phage ϕAB2 (10^9^–10^10^ plaque-forming units (PFU)/ml) was prepared via plate lysis and elution. ϕAB2 was propagated and assayed in triplicate using the double-agar-layer method as previously described [45].

### Phage adsorption assay

*A. baumannii* M3237 was infected with phage ϕAB2 at a multiplicity of infection (MOI; phage concentration/bacterial concentration) of 0.001 and incubated at room temperature. The bacterial host *A. baumannii* ATCC 17978 was also evaluated for comparison. Samples (100 μl) were taken at 2-min intervals for 10 min, diluted in 0.9 ml of cold LB, centrifuged (12,000 × *g*, 5 min), and supernatants containing unadsorbed phages were titrated.

### Effect of temperature on ϕAB2 stability

ϕAB2 stock (10^10^ PFU/ml) was diluted to 10^8^ PFU/ml with distilled water. The mixed phage solution was subsequently divided into 1 ml vials and stored at −20°C, 4°C, or 25°C. At various time points up to 360 days, solution from one vial at each temperature was inoculated for plaque assay. Used vials were discarded. To assess the effect of refreezing on phage survival, a vial with 500 ml of a 10^8^ PFU/ml phage solution was stored at −20°C and inoculated for plaque assays at various time points, after which the solution was stored at −20°C again until the next sampling time.

### Effect of pH on ϕAB2 stability

The stability of ϕAB2 at different pH values was determined by mixing 10^10^ PFU/ml of ϕAB2 suspension with sterile water at different pH values (pH 2, 4, 7, or 11) to obtain a 100 ml phage solution with a final phage concentration of 10^8^ PFU/ml. The pH was adjusted with 1 N HCl or KOH. After phage solutions were prepared, the initial concentration was determined within 5 min, and then stored at 25°C until used.

### Effect of chloroform concentration on ϕAB2 stability

Briefly, phage solutions (10^8^ PFU/ml) were exposed to 0.5% or 2% chloroform. The first sample was inoculated within 5 min to determine the initial concentration, and the solution was then inoculated for plaque assays at different storage times up to 360 days.

### Stability of ϕAB2 on glass slides

Aliquots of 100 μl of a 10^9^ PFU/ml ϕAB2 suspension were spiked on the surface of sterilized glass slides (10^8^ PFU/13.8 cm^2^ surface), and incubated in a biosafety hood at room temperature for 30 min until completely dry. At various time points, a spiked glass slide was placed into a conical tube with 20 ml of peptone and gently vortexed for 30 s. ϕAB2 recovered in the eluant was enumerated by plaque assay. All desiccation experiments were performed at room temperature (25°C) and 55–65% relative humidity.

### Bactericidal effect of ϕAB2 in a liquid suspension

To determine the bactericidal effect of ϕAB2 in suspension, *A. baumannii* M3237 was cultured overnight and then transferred to a flask and incubated at 37°C until reaching an OD_600_ of 1.0 (5 × 10^8^ CFU/ml). *A. baumannii* M3237 cultures were then serially diluted to obtain final concentrations of 5 × 10^6^, 5 × 10^5^ or 5 × 10^4^ CFU/ml. A 1 ml aliquot of each concentration was mixed with 1 ml of ϕAB2 suspension to obtain a final phage concentration of 10^3^, 10^5^, or 10^8^ PFU/ml. Phage-free culture (containing bacteria only) was included as a control. Following a 5- or 10-min incubation, host and phage mixtures were immediately passed through 47-mm diameter membrane filters (pore size of 0.45 μm, Pall Corporation) and washed with 20 ml of phosphate-buffered saline (PBS) to remove unattached phages [26]. Washed filters were placed in separate dishes containing LB agar, and following 24-h incubation at 37°C, the number of recovered *A. baumannii* M3237 was calculated by counting colonies on each filter. The survival rate was calculated as log_10_ of N_t_/N_0_, where N_0_ is the number of *A. baumannii* M3237 colonies recovered on the control filter and N_t_ is the number of colonies on the test filter.

### Bactericidal effect of ϕAB2 on a glass slide

To determine the bactericidal effect of ϕAB2 on a glass surface, glass slides were sterilized, pre-contaminated with *A. baumannii* M3237 by spreading diluted culture stock solution on the glass surface to obtain concentrations of 10^4^, 10^5^, and 10^6^ CFU/slide and dried for 30 min in a biosafety hood at room temperature. Then, slides were divided into two groups. 1) test: treated with ϕAB2 to reach a concentration of 10^3^, 10^5^, or 10^8^ PFU/slide and 2) control: treated with phage-free suspension. After the ϕAB2 solution or phage-free suspension was applied to the *A. baumannii* M3237 slide, they were stored for 5 or 10 min at room temperature. Residual *A. baumannii* M3237 particles on the test or control slides were eluted with 20 ml of peptone into a conical tube, gently vortexed for 30 s, serially diluted and passed through membrane filters, as above. The filters were then washed with PBS, placed on LB agar plates, and incubated for 24 h at 37°C. The number of *A. baumannii* M3237 colonies that grew on each filter was counted and the survival rate was calculated.

### Production of ϕAB2 hand sanitizer in a paraffin oil-based lotion

A commercial cream containing paraffin mineral oil (First Chemical Works, Taipei, Taiwan) was combined with ϕAB2 in a conical tube and sterile water added to obtain a paraffin oil-based lotion with a final concentration of 10% (v/v) paraffin oil and a phage concentration of 10^8^ PFU/ml. The phage-containing lotion was stored at room temperature up to 30 days. At each sampling point, the phage lotion was inoculated for plaque assays to obtain a kinetic curve of the phage concentration.

The relationship between the number of residual ϕAB2 phages in the lotion and their ability to inhibit MDRAB was evaluated. At each sampling point, LB agar was pre-contaminated with *A. baumannii* M3237 suspension to obtain surface concentrations of 5 × 10^1^, 5 × 10^2^, and 5 × 10^3^ CFU/ml. Contaminated agar plates were dried for 30 min in a biosafety hood at room temperature and divided into two groups: test agars received 0.1 or 0.5 ml of the phage-containing lotion to simulate the volumes of lotion used by most hand cream consumers and control. The control agars consisted of a phage-free lotion. The test and control agars were then incubated for 24 h at 37°C, and bacterial recovery counts calculated by comparing the number of *A. baumannii* M3237 colonies from the test agars with those from the control agars.

### ϕAB2 in glycerol as a hand sanitizer

Briefly, the phage stock was mixed with glycerol to obtain a solution of 10% (v/v) glycerol/10^8^ PFU/ml phage and stored at room temperature for up to 180 days to obtain a kinetic curve of the phage variation during this period. Phage stability and ability to inhibit *A. baumannii* M3237 was determined as described above for lotions.

### Statistical analysis

Statistical analyses were performed using SPSS, version 17.0 (SPSS Institute Inc., Chicago, IL, USA). Measurement of ϕAB2 bactericidal effect in liquid suspensions and glass slides, comparison of *A. baumannii* M3237 survival rates with different incubation times and control sets and reduction of viable *A. baumannii* M3237 by ϕAB2 lotion or glycerol was performed using one-way ANOVA, followed by Tukey’s test.

## Competing interests

The authors declare that they have no competing interests.

## Authors’ contributions

LKC and YLL performed the experiments and analyses. AH and KCC provided test materials and participated in the analysis of bacteria. NTL and MJL participated in the bacteriophage experiments. CCT conceived of the study and drafted the manuscript. All authors read and approved the final manuscript.
